# Where True Quality Is Shown

**Published:** 1905-03

**Authors:** 


### WHERE TRUE QUALITY IS SHOWN.

The excellence of Scott's Emulsion is recognized by the highest authority. The *London Lancet* said of it: "The value of the hypophosphites combined with cod-liver oil, especially in wasting diseases and debilitated conditions, is well known. In addition to these constituents, Scott's Emulsion also contains glycerine, which is well recognized as assisting very materially in the absorption of oils and fats. We have examined the preparation with care, and find that it fulfills all the requirements and presents all the conditions of a very satisfactory emulsion. In appearance and consistence it is not unlike cream, and under the microscope the fat globules are seen to be of perfectly regular size and uniformly distributed. In fact, the preparation, microscopically examined, presents the appearance of cream. So well has the oil been emulsified that even when shaken with water the fat is slow to separate, the liquid then looking like milk. The taste is decidedly unobjectionable and is pleasantly aromatic and saline. We had no difficulty in recognizing the presence of the hypophosphites in an unimpaired state. The Emulsion keeps well even when exposed to wide changes of temperature. Under the circumstances just described the Emulsion should prove an excellent food as well as a tonic."

---

The number of registered physicians in the Russian empire is now 21,827, of whom 737 are women. All but 2,659 reside in European Russia. Siberia has 788, the Caucasus district 1,224 and the Central Asia possessions 459. The last restrictions have been removed from the medical career for women, and the graduates of the woman's medical college at St. Petersburg now rank in every respect with the graduates of the other medical schools of the empire.—*Medical Review of Reviews.*

---

A tax assessor by "pull" gave a citizen the surprising tax of $24 on personal property consisting of one goat. In reply to remonstrance, the following construction of the law was rendered: "Here is what the law says: 'For all property bounding or abutting on the highway, $12 per front foot; and two fore feet foot up $24.'"

